# Lipoprotein Lipase Is a Feature of Alternatively-Activated Microglia and May Facilitate Lipid Uptake in the CNS During Demyelination

**DOI:** 10.3389/fnmol.2018.00057

**Published:** 2018-03-15

**Authors:** Kimberley D. Bruce, Sachi Gorkhali, Katherine Given, Alison M. Coates, Kristen E. Boyle, Wendy B. Macklin, Robert H. Eckel

**Affiliations:** ^1^Division of Endocrinology, Metabolism, & Diabetes, Department of Medicine, University of Colorado Denver Anschutz Medical Campus, Aurora, CO, United States; ^2^Department of Cell and Developmental Biology, University of Colorado School of Medicine, Aurora, CO, United States; ^3^School of Health Sciences, Sansom Institute for Health Research, University of South Australia, Adelaide, SA, Australia; ^4^Department of Pediatrics, Section of Nutrition, University of Colorado School of Medicine, Aurora, CO, United States

**Keywords:** lipoprotein lipase (LPL), microglia, multiple sclerosis, myelination, lipid metabolism

## Abstract

Severe demyelinating disorders of the central nervous system (CNS) such as multiple sclerosis (MS), can be devastating for many young lives. To date, the factors resulting in poor remyelination and repair are not well understood, and reparative therapies that benefit MS patients have yet to be developed. We have previously shown that the activity and abundance of Lipoprotein Lipase (LPL)—the rate-limiting enzyme in the hydrolysis of triglyceride-rich lipoproteins—is increased in Schwann cells and macrophages following nerve crush injury in the peripheral nervous system (PNS), suggesting that LPL may help scavenge myelin-derived lipids. We hypothesized that LPL may play a similar role in the CNS. To test this, mice were immunized with MOG_35–55_ peptide to induce experimental allergic encephalomyelitis (EAE). LPL activity was increased (*p* < 0.05) in the brain at 30 days post-injection, coinciding with partial remission of clinical symptoms. Furthermore, LPL abundance and activity was up-regulated (*p* < 0.05) at the transition between de- and re-myelination in lysolecithin-treated* ex vivo* cerebellar slices. Since microglia are the key immune effector cells of the CNS we determined the role of LPL in microglia. Lipid uptake was decreased (*p* < 0.001) in LPL-deficient BV-2 microglial cells compared to WT. In addition, LPL-deficient cells showed dramatically reduced expression of anti-inflammatory markers, YM1 (−22 fold, *p* < 0.001), and arginase 1 (Arg1; −265 fold, *p* < 0.001) and increased expression of pro-inflammatory markers, such as iNOS compared to WT cells (+53 fold, *p* < 0.001). This suggests that LPL is a feature of reparative microglia, further supported by the metabolic and inflammatory profile of LPL-deficient microglia. Taken together, our data strongly suggest that LPL expression is a novel feature of a microglial phenotype that supports remyelination and repair through the clearance of lipid debris. This mechanism may be exploited to develop future reparative therapies for MS and primary neurodegenerative disorders (Alzheimer’s disease (AD) and Parkinson’s disease).

## Introduction

Multiple sclerosis (MS) is the most common cause of neurological disability in young adults, affecting approximately 2.3 million people worldwide. The majority of MS patients show a relapsing-remitting (RR-MS) clinical phenotype, in which oligodendrocyte precursor cells (OPCs) attempt to remyelinate areas of myelin damage. However, as the disease progresses these attempts fail, leading to severe secondary progressive MS (P-MS; Antel et al., 2012). Despite recent advances in our understanding of the pathophysiology of MS, the factors resulting in poor remyelination and repair are not well understood. Consequently, much needed reparative therapies that benefit MS patients have yet to be developed.

Microglia are the key immune effector cells of the central nervous system (CNS), where they have essential functions such as synaptic pruning by engulfment of neuronal terminals (Hong and Stevens, 2016), and phagocytosis of dying cells and myelin debris (Ransohoff and Perry, 2009). CNS microglia have been repeatedly implicated in the pathophysiology of MS (Ransohoff and El Khoury, 2015). On one hand, CNS microglia may be considered “classically activated” and be associated with the production of neurotoxic molecules and pro-inflammatory cytokines that are detrimental to the repair processes (Becher et al., 2001; Heppner et al., 2005). In contrast, “alternatively activated” reparative microglia provide neurotrophic and immunosuppressive factors that counteract pathological processes and may help facilitate remyelination (Schwartz and Moalem, 2001; Hohlfeld, 2008). Although the plethora of intermediate microglial phenotypes associated within a disease process make it difficult to categorize microglial sub-populations into defined activation states, it is a useful starting point to investigate the basic mechanisms by which microglia participate in demyelination and remyelination. In MS, appropriately activated microglia may help clear debris after myelin damage (Lampron et al., 2015), expedite extracellular matrix deposition, facilitate regeneration, and offer trophic support to OPCs (Varin and Gordon, 2009). In support of this notion, when debris clearance by microglia is impeded, regeneration is delayed (Neumann et al., 2009). However, the microglial factors that actively facilitate these processes are unknown.

In peripheral tissues such as adipose tissue and skeletal/cardiac muscle the canonical function of lipoprotein lipase (LPL)—hydrolysis of triglyceride-rich lipoproteins—is well defined. However, the role of LPL in cells of the CNS is not well understood. Nonetheless, previous work from our laboratory and others have shown that LPL is expressed in several regions of the nervous system including the brain, spinal cord and peripheral nerves, with the highest expression observed in the hippocampus and spinal cord (Eckel and Robbins, 1984; Goldberg et al., 1989; Bessesen et al., 1993). Our studies show that in the peripheral nervous system (PNS), LPL is expressed in Schwann cells, macrophages, and dorsal root ganglia neurons, and its abundance and activity is increased following nerve crush injury (Huey et al., 2002; Nunes and Sousa, 2008). This increase in LPL is associated with macrophage infiltration, suggesting that LPL may be part of an acute response to scavenge and reutilize myelin-derived lipids in degenerating peripheral nerves (Huey et al., 2002). However, this has yet to be empirically determined.

A number of recent reports have highlighted LPL as a key feature of reparative CNS microglia. Single cell analysis of disease-associated-microglia (DAM) in a murine model of Alzheimer’s disease (AD), shows that LPL is markedly increased in a unique microglial sub-type associated with phagocytosis and protection in advanced stages of AD (Keren-Shaul et al., 2017). In addition, in a cuprizone model of demyelination, LPL gene expression was elevated during demyelination, and tailed-off once remyelination was complete, suggesting that LPL is novel feature of a microglial phenotype that actively supports remyelination and repair (Olah et al., 2012). Despite these important observations the mechanisms underlying the role of LPL in CNS microglia have not been determined.

Here, we hypothesize that LPL may support repair through the clearance of myelin-derived lipids. In the current study we utilize *in vivo*, *ex vivo* and *in vitro* systems to demonstrate that LPL is increased during the onset of remyelination, is associated with an anti-inflammatory reparative microglial phenotype, and may facilitate the uptake of myelin-derived lipids in the CNS.

## Materials and Methods

### Animals

This study was carried out in accordance with University of Colorado Institutional Animal Care and Use Committee (IACUC) guidelines IACUC for animal use, which are in agreement with the NIH Guide for the Care and Use of Laboratory Animals. Animal protocols were approved by the University of Colorado IACUC. Male C57Bl/6J wild-type mice 8–10 weeks-old were obtained from Jackson Labs (Bar Harbor, Maine, ME, USA). Mice were individually housed and maintained at ~20°C with a 12-h light/dark cycle and given unrestricted access to standard laboratory diet (Diet 8640; Harlan Teklad, Madison, WI, USA) and water. Paralyzed mice were afforded easier access to food and water to prevent dehydration.

### Antigens

The peptide used in this study was the immunodominant MOG_35–55_ peptide (MEVGWYRSPFSRVVHLYRNGK; Mendel et al., 1995). The purity was assessed by HPLC (>97% pure) and amino acid composition was verified by mass spectrometry (Peptides International, Louisville, KY, USA). MOG_35–55_ peptide batches for *in vivo* work were all from one preparation. They were stored insoluble until required and then dissolved in saline to a concentration of 150 μg/μl. The unused portions were stored at 4°C for a maximum of 3 months.

### Induction of Experimental Autoimmune Encephalitis (EAE)

Mice were injected subcutaneously at two sites on the femoral region with 200 μl of a mixture of MOG _35–55_ peptide emulsified 1:1 with Freund’s complete adjuvant (Difco Laboratories, Detroit, MI, USA). MOG treated mice were also boosted with pertussis toxin (List Laboratories, Campbell, CA, USA; 200 ng) I.P. on both the day of injection and also 48 h later. Control mice were injected with equal volumes of CFA and given saline instead of pertussis toxin. Tissues were harvested at days 10, 20, 30 and 60 post injection after the animals had been fasted for 4 h before being anesthetized with Avertin (2,2,2-tribromoethanol, 32 mg; Aldrich, Milwaukee, WI, USA).

### Clinical Evaluation

Individual mice were observed daily for clinical signs of disease for up to 60 days after immunization. Mice were scored according to the following scale: 0, no detectable signs of experimental allergic encephalomyelitis (EAE); 0.5, incomplete tail paralysis; 1, complete limp tail; 2, hind limb weakness and unsteady gait; 3, complete hind limb paralysis; 4, total paralysis of both forelimbs and hind limbs; 5, moribund; 6, death (Okuda et al., 2002). Body weights and food intake was also measured daily. The data were plotted as daily mean clinical score for all animals in a particular treatment group.

### General Cell Culture Conditions and Reagents

Immortalized BV-2 murine microglia cells were kindly donated by Dr. Peter Grace (University of Colorado, Boulder). BV-2 cells were grown in Dulbecco’s modified Eagle’s medium (DMEM) containing 10% fetal bovine serum (FBS) and 1% penicillin/streptomycin (P/S) and maintained at 37°C with 5% CO_2_ (unless otherwise stated). Cells were grown to 70%–90% confluency for all experiments (unless otherwise specified).

### LPL Targeting by shRNA

LPL was knocked down (KD) in BV-2 microglia using shRNA targeting, as previously described (Libby et al., 2015). In brief, for stable KD of LPL, 12 μg of control (202) or LPL shRNA (553) lentiviral vector was transfected into HEK 293FT cells along with 10.8 μg pΔ8.9 and 1.2 μg VSV-G using 45 μL Lipofectamine 2000. Virus was packaged for 48 h in 6 mL media, and two rounds of BV-2 transduction were performed. Stably-transduced cells were then selected for 3 days by growth in medium containing 5 μg/mL puromycin.

### Real-Time RT PCR

Total RNA was isolated from BV-2 cells using RNeasy Plus Mini Kit (Qiagen, Valencia, CA, USA) according to the manufacturer’s instructions. cDNA was prepared from 1 μg of total RNA using verso cDNA synthesis kit (Thermo Scientific, Lithuania). Relative gene expression levels quantified by real-time PCR using SYBR Select Master Mix (Applied Biosystems, Foster City, CA, USA). Primers were designed using the NCBI primer-blast tool and specific primers were designed to cross exon boundaries where available. The sequences for all primers used in the study are shown in Table 1. All PCR was performed with the following cycling conditions: 50°C for 2 min, 95°C for 10 min, followed by 40 cycles of 95°C for 15 s and 60 for 1 min using the StepOnePlus instrument (Applied Biosystem, Foster City, CA, USA) with complementary StepOne™ Software v2.3. Comparative Ct method was used to determine relative target gene expression and data were normalized to an average of the references genes Ubc and GAPDH.

**Table 1:** Primer Sequences.

Gene	Accession	Forward	Reverse
LPL	NM_008509	ATGGATGGACGGTAACGGGAATGT	TGGATAATGTTGCTGGGCCCGATA
Arg1	NM_007482.3	GGTCTGTGGGGAAAGCCAAT	AACTGCCAGACTGTGGTCTC
YM1	NM_009892.3	GTACCCTGGGTCTCGAGGAA	CCTTGGAATGTCTTTCTCCACAG
iNOS	NM_010927.4	GGTGAAGGGACTGAGCTGTT	ACGTTCTCCGTTCTCTTGCAG
IGF-1	NM_010512.5	CTCAGAAGTCCCCGTCCCTA	ATTTTCTGCTCCGTGGGAGG
CCL3	NM_011337.2	CCATATGGAGCTGACACCCC	TCAGGAAAATGACACCTGGCT
IL-6	NM_031168.2	CTCTGCAAGAGACTTCCATCCA	GACAGGTCTGTTGGGAGTGG
IL-12	NM_001159424.2	TTCTCACCGTGCACATCCAA	GAGGAGGTAGCGTGATTGACA
MSR1	NM_031195.2	GGGAGTGTAGGCGGATCAAG	ATAGTAGGGTGCTCTGCCCA
CD36	NM_001159558.1	AGAATTCTCAGCTGCTCCGC	ACACATTTCAGAAGGCAGCAAC
VLDLR	NM_013703.2	TCAACTGCCCTTCTCGAACC	AGCCATCAACACAGTCTCGG
LDLR	NM_010700.3	CCAATCGACTCACGGGTTCA	TCACACCAGTTCACCCCTCT

### Western Blots

BV-2 cells were grown to 90% confluency and total protein was harvested using RIPA buffer (150 nM NaCl, 1% Triton X-100, 0.5% Sodium Deoxycholate, 0.1% SDS, 50 mM Tris pH 8.0, and Complete Protease Inhibitor Cocktail). Insoluble cell debris were removed by centrifugation, and protein containing supernatants were retained. Protein quantification was performed using the Peirce™ BCA protein Assay kit (Thermo). Twenty-five microgram of total protein extracts were run on 12% Separating Gel and transferred to nitrocellulose membrane. The membrane was blocked in skim milk and incubated with a proprietary anti-mouse anti-LPL primary antibody raised in rabbit at a concentration of 3 μg/ml 397-1 (397-1, NeoBiolab, Cambridge, MA, USA) and 1 μg/ml mouse anti-GAPDH (MAB374, Millipore, Billerica, MA, USA) overnight at 4°C. Membranes were then incubated with secondary antibodies IRDye 800RD goat anti-rabbit and IRDye 680RD donkey anti-mouse (Li-Cor, Nebraska, NE, USA) at 1:10,000 dilution. Protein bands were visualized using the Li-COR Odyssey with complementary software (Image studio).

### LPL Enzymatic Activity

The enzymatic activity of cell-surface heparin-released LPL was measured using a phospholipid (PL)/^3^H-triolein substrate with human serum as a source of ApoC2 as described previously (Jensen et al., 2008).

### LPS Stimulation

BV-2-553 and BV-2-202 and primary adult microglia were stimulated with 1 μg/ml lipopolysaccharide (LPS) in DMEM containing 1% FBS and 1% P/S media for 24 h. Gene expression and LPL activity assays were then conducted.

### Lipid Uptake Experiments

Lipid Uptake Experiments were performed as previously described (Libby et al., 2015), with limited modifications. The assay was performed by preparing a synthetic PL/TG emulsion containing a ^3^H triolein tracer. The emulsion was prepared by combining and sonicating the following: 5 mg ^1^H triolein, 0.25 mg L-phosphatidylcholine, a trace amount of ^3^H triolein (12.5 μCi total) tracer (NET431001MC, Perkin Elmer, Waltham, MA, USA), 0.9 mL water, 2 mL 1 M tris–HCl (pH 8.0), 800 μL FFA-free BSA (MP Biomedical), and 300 μL KRP. After sonication, the substrate was diluted into DMEM to a final concentration of 50 μM, and 100 μM triolein. Purified human ApoC2 (MyBioSource, SanDiego, CA, USA) was added to a final concentration of 5 μg/mL. Substrate was applied to over-night serum-starved BV-2-553 and BV-2-202 cells for 2 h. Cells were washed with 0.1% FFA-free BSA and lysed in 1 mL of RIPA buffer. Eight-hundred microliter of each lysis fraction was subjected to scintillation counting, while the rest was used for protein normalization.

### Uptake of DiI Labeled Liposomes

Synthetic PL/TG chylomicrons were prepared as described above in the absence of radiolabeled triolein. PL containing vesicles were prepared by drying an 80:20 molar ratio of phosphatidylcholine (PC, Avanti) and phosphatidylserine (PS, Avanti) under nitrogen. HBS was added to the dried lipid for 1 h prior to sonication. Ten microliter of a 3 mg/ml solution of 1′-dioctadecyl-3,3,3′3′-tetramethylindocarbocyanine perchlorate (DiI) was added to 1 ml of either SC’s or PL vesicles and incubated in the dark for 10 min at 37°C. Free label was removed with ultracentrifugation at 24,000 *g* for 10 min. After resuspension in sterile PBS, labeled liposomes were added to cells for 16 h (overnight) at a final concentration of 250 μg/ml.

### Conditioned Media Experiments

Conditioned media (CM) was harvested from either BV-2-553 or BV-2-202 cells grown in DMEM containing 1% FBS for 72 h reaching 90% confluency. Following aspiration, the media was briefly centrifuged to remove cellular debris, and media was either used immediately for experiments or flash frozen and stored at −80°C.

### Neuro-Discovery Array

Semi-quantitative analysis of cytokines and cell signaling proteins from BV-2-CM was performed using the RayBio^®^ C-series Mouse Neuro Discovery Array C1 (RayBiotech, Norcross, GA, USA) according to manufacturer’s instructions. In brief, membranes were incubated with 1 ml of CM (described above) overnight at 4°C, while the biotinylated antibody and streptavidin incubation steps were performed at 2 h at room temperature (RT). The array was then visualized using chemiluminescence detection.

### Cerebellar Slice Culture, Lysolecithin and Conditioned Media Treatment

Sagittal cerebellar slices (300 μm) were prepared from PLP-eGFP mice (Mallon et al., 2002) at P10–P12 and cultured on MilliCell 0.4 μm membrane inserts (Millipore, Billerica, MA, USA) for 7 days in slice media (25% HBSS, 25% heat-inactivated horse serum, 50% MEM, 125 mM HEPES, 28 mM D-Glucose, 2 mM L-Glutamine; Sheridan and Dev, 2012). Slices were incubated at 37°C at 5% CO_2_ and slice media was changed within 24 h after preparation, then every 3–4 days following. Lysolecithin (Lyso; Sigma) was prepared as a 5 mg/ml stock and stored at −20°C until use. To prepare a working solution, lysolecithin stock was diluted in slice media at 0.5 mg/ml and sterile filtered. Treatments (Slice media or lysolecithin) were applied both on top (50 μl) and below (250 μl) the membrane insert that contained the slice for 17 h at 37°C at 5% CO_2_. After 17 h, slices were washed twice with slice media and then incubated in control media or CM from BV-2 microglia.

### Slice Fixation and Immunostaining

After treatment, cerebellar slices were rinsed twice with PBS and fixed in 4% paraformaldehyde in PBS for 20 min. For immunohistochemistry, slices were rinsed in PBS and permeabilized in 1.5% or 10% (for myelin proteins) Triton X-100 in PBS for 20 min. Slices were rinsed, blocked with 5% normal donkey serum (NDS) in PBSTx (0.3%) for 1 h, and incubated with primary antibodies overnight at RT. Following three washes in PBS, secondary antibodies (Jackson ImmunoResearch) were applied 1:800 overnight at RT, slices were washed three times in PBS and mounted in Fluoromount G (Southern Biotech). The following primary antibodies were used: Rb α glial fibrillary acidic protein, GFAP (Sigma, G3893), Rb α Calbindin (Millipore, AB1778), Ms α myelin-associated glycoprotein, Myelin associated glycoprotein (MAG; Millipore, MAB1567), Rb α myelin-oligodendrocyte glycoprotein, MOG (Abcam, ab32760), Ms α myelin basic protein, MBP (Covance, SMI 94), Ckn α Neurofilament-H (Neuromics, CH22104), Gt α Iba1 (Abcam, ab5076), Rb α NG2 proteoglycan, GP α NG2 proteoglycan (gifts from Dr. William Stallcup, Burnham Institute), and Rb α Olig2 (a gift from Dr. Charles Stiles, Harvard University).

### Immunocytochemistry of BV-2 Cells

BV-2-202 and BV-2-553 cells were grown to about 40% confluency on a coverslip. Media was then replaced with CM from BV-2-202 cells, BV-2-553 cells or regular control media for 24 h. Cells were fixed in 4% paraformaldehyde for 20 min at RT and incubated with 1 μg/ml of rabbit anti-Iba1 primary antibody (Wako, Osaka, Japan) overnight at 4°C. Cells were then incubated in Alexa Fluor^®^ goat anti-rabbit 488 (Molecular Probes, Eugene, OR, USA) secondary antibody at 1:400 for 1 h at RT. Vectashield mounting media with DAPI (Vector Laboratories, Inc., Burlingame, CA, USA) was used to mount coverslip onto slide and stain nuclei. Confocal laser scanning microscopy (Olympus FV1000, Olympus, Tokyo, Japan, using Olympus Fluoview Software), was used to image the cells. Exposure parameters were identical in all slides.

For DiI labeled lipid uptake experiments the average fluorescence per cell was determined by measuring the mean fluoresce in *N* = 10 induvial cells from each experimental group using the cell magic wand plugin for ImageJ.

### Primary Microglia Isolation

Primary microglia were isolated from adult brains of Cx3CR1^CreER^ (Jackson Labs) which express a Cre-ERT2 and EYFP in microglia as previously described with very limited modifications (Lee and Tansey, 2013). Care was taken to dissociate cells with large, medium and small-sized hole polished Pasteur pipettes.

### Immunohistochemistry of Primary Microglial Cells

Primary microglia cells were plated on poly-D-lysine coated glass coverslips and cultured for 2 days. Cells were washed with HBSS 1× and fixed in 4% paraformaldehyde at RT for 20 min. Cells were blocked for 1 h with 10% normal goat serum (NGS) in PBST. Cells were incubated with 1 μl Anti-Iba1, rabbit (Wako) for 16 h at 4°C. The cells were washed 3× with PBS. Secondary goat anti-rabbit 488 was diluted 1:400, cells were incubated for 1 h at RT. Coverslips were mounted with Vectashield Anti Fade Mounting Media with DAPI, and slides were imaged using confocal Olympus FV1000.

### Metabolomics

Cell pellets (10^6^ cells) were stored at −80°C and were extracted in ice-cold lysis/extraction buffer (methanol:acetonitrile:water 5:3:2 v/v). Amino acids (Nemkov et al., 2017), and other metabolites (Nemkov et al., 2015), were quantified in cell extracts as previously described.

### Fatty Acid Oxidation (FAO) Assays

Fatty Acid Oxidation (FAO) assays were performed as described with limited modifications (Boyle et al., 2017). Control and LPL KD BV-2 microglia were incubated at 37°C in sealed 24-well plates containing differentiation media containing 0.25 μCi/ml [^14^C]oleate and 0.25 μCi/ml [^14^C]palmitate (PerkinElmer Life Sciences, Waltham, MA, USA), with 200 μM, 1:1 “cold” oleate:palmitate. After a 2 h incubation, the rate of FAO was determined by measuring the ^14^CO_2_ released from the media following acidification. Measures were performed in quintuplet and data were corrected for total protein content, determined by BCA assay (Pierce Biotechnology, Inc., Waltham, MA, USA).

### Statistical Analysis

The difference between mean values for each experimental group were determined by either an ANOVA (for three or more experimental groups) followed by *post hoc* analysis with Bonferroni correction for multiple comparisons, or unpaired *t*-test (for experiments with two experimental groups), since data showed Gaussian distribution.

## Results

### LPL Activity Is Increased at the Interphase of De- and Remyelination

**Figure 1:**
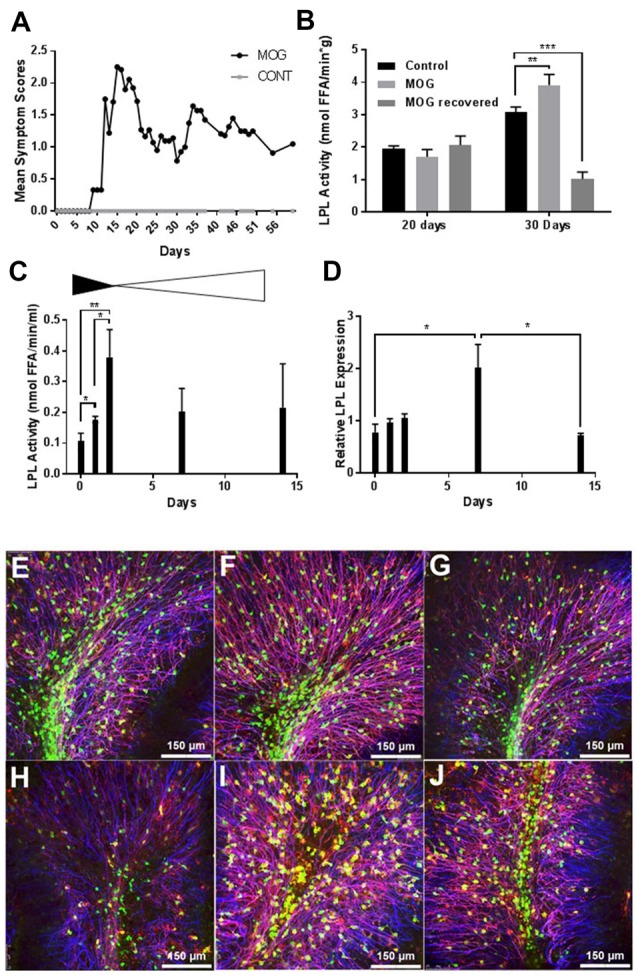
Lipoprotein lipase (LPL) is increased during de- and re-myelination. **(A)** Mean symptom scores of MOG treated (*N* = 10) and Control (*N* = 20) mice**. (B)** LPL activity in brain tissue at 20 and 30 days MOG post injection from Control (*N* = 20), MOG-treated (symptomatic; *N* = 10), and MOG-recovered (MOG-treated but asymptomatic following an initial bout of symptoms, *N* = 3)**. (C)** LPL enzymatic activity in cerebellar slice cultures at 0, 1, 2, 7 and 14 days post LPC treatment (*N* = 3 per group, per time point). **(D)** LPL gene expression in cerebellar slice cultures at 0, 1, 2, 7 and 14 days post LPC treatment (*N* = 3 per group, per time point). **(E–J)** Lysolecithin-mediated demyelination of* ex vivo* cerebella brain slices (*N* = 3 per group, per time point). Blue—neurofilament (NFH), Green—PLPeGFP, Red—myelin associated glycoprotein (MAG). **(E–G)** No Lysolecithin control, 1, 4 and 7 days. **(H–J)** 1, 4 and 7 days post Lysolecithin treatment. *P < 0.05 vs. CONT (at corresponding time point). **P < 0.01 vs. CONT (at corresponding time point). ***P < 0.001 vs. CONT (at corresponding time point).

EAE is a CNS autoimmune disease that has been used to investigate human demyelinating diseases such as MS (Johns et al., 1995). EAE has been well characterized in rodents using both recombinant and synthetic myelin proteins including MOG or MOG peptides (Amor et al., 1994; Johns et al., 1995; Frausto et al., 2007). To induce EAE we chose the MOG_35–55_ peptide as a suitable antigen since MS patients predominantly generate anti-MOG antibodies (Goudonnet et al., 1990). Following immunization with MOG_35–55_ or vehicle_,_ mice were scored daily for severity of clinical symptoms. By 20 days post immunization MOG_35–55_ mice had symptoms ranging from a limp tail (score of 1) to completely paralyzed hind limbs (score of 3) while the control mice showed no symptoms of paralysis. The control mice continued to show no signs of paralysis from 1 day through to 60 days after treatment while the MOG-treated mice had relapsing remitting symptoms (Figure 1A). At 20 days (when clinical symptoms were at their peak in the MOG group) and 30 days (when clinical symptoms reached a nadir in the MOG group), LPL activity was measured in whole brain tissue (Figure 1B). Interestingly, while there was no difference in LPL activity in the brain at 20 days, LPL activity was increased in the brain by 30 days (*P* < 0.01), suggesting that increased LPL activity was associated with the initiation of improved symptoms.

To further understand the dynamics of LPL function during active demyelination and subsequent remyelination, we measured LPL enzymatic activity and expression in lysolecithin-treated *ex vivo* organotypic slice cultures of mouse cerebellum. The *ex vivo* slice culture system closely resembles the *in vivo* environment of the CNS, with the added benefit of ease of high throughput experimental treatments (Harlow et al., 2015). In this system, short term treatment with lysolecithin produces dramatic demyelination in brain slices *ex vivo* (Birgbauer et al., 2004). Twenty-four hours after lysolecithin removal there is continued demyelination and a significant reduction of the myelin markers, MBP, MOG and cyclic nucleotide phosphodiesterase (CNPase; Birgbauer et al., 2004). Several days after lysolecithin treatment these markers began to increase, concomitant with the initiation of remyelination. Here we show that heparin-releasable LPL enzymatic activity from the surface of cerebellar slices was significantly increased after 2 days of recovery in normal medium (Figure 1C), suggesting that LPL is most active and abundant at the end of demyelination and the initiation of remyelination. In addition, LPL expression was significantly increased by 7 days post-lysolecithin treatment (Figure 1D), which is consistent with the recovery of myelin markers, MAG and proteolipid protein1 (PLP1) by 7 days (Figures 1E–J).

### The Loss of LPL in BV-2 Microglia Polarizes BV-2 Microglia to a Pro-inflammatory Phenotype

**Figure 2:**
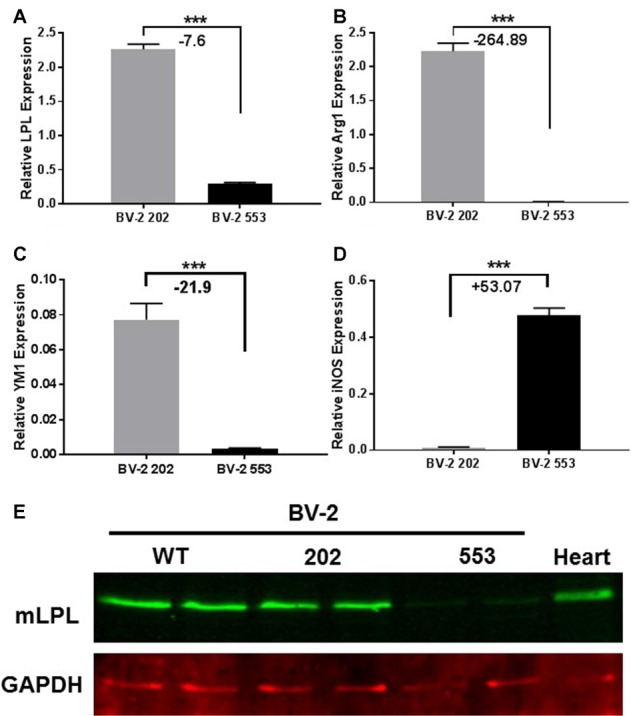
Characterization of LPL knock down (KD) cells. **(A–D)** Gene expression in microglial BV-2 KD (553) vs. control (202) cells (*N* = 4 per group). **(E)** Western blot analysis showing reduced LPL protein expression in BV-2 553 cells. ***P < 0.001 vs. CONT.

Since CNS microglia have been repeatedly implicated in myelination dynamics (Sun et al., 2017), and recent reports have highlighted the pivotal role of microglial LPL in remyelination and primary neurodegenerative disorders (Olah et al., 2012; Keren-Shaul et al., 2017), we utilized BV-2 microglia to investigate the role of LPL within these cells. BV-2 cells are an immortalized mouse microglial cell line that retains many of the morphological and functional characteristics of microglia, with the ability to stably alter the levels of LPL expression and activity (Blasi et al., 1990; Bocchini et al., 1992; Wen et al., 2006; Fu et al., 2017). We stably KD LPL expression in BV-2 cells using an LPL shRNA lentiviral vector, previously validated in our laboratory, to generate BV-2 553 (LPL KD) cells (Libby et al., 2015). An empty lentiviral vector was used to generate BV-2 202 (control) cells. Comparative gene expression between LPL KD and control cells showed a dramatic decrease in LPL KD cells in the expression of genes classically associated with an “M2” or “alternatively activated” phenotype. For example, the enzyme arginase 1 (Arg1), which converts arginine to polyamines and contributes to wound healing and repair (Munder, 2009; Cherry et al., 2014), showed a −265-fold reduction in LPL KD cells (Figure 2B). Similarly, chitinase-like 3 (YM1), a secretory lectin that binds heparin sulfate and prevents degradation of extracellular matrix proteins showed a −22-fold reduction in gene expression in LPL KD cells (Figure 2C). In contrast, iNOS expression, commonly associated with an M1-like microglial phenotype was markedly elevated in LPL KD cells (+53 fold; Figure 2D). Taken together, these data strongly suggest that microglial LPL expression is a feature of alternatively activated microglia.

**Figure 3:**
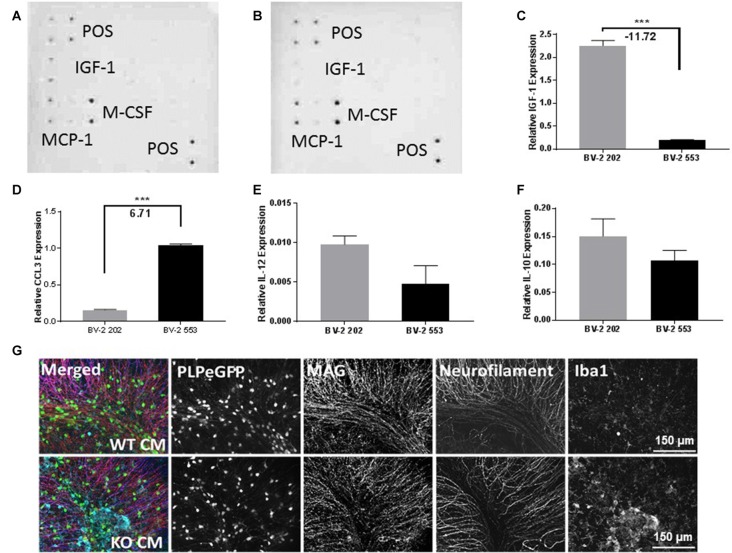
Chemokine and cytokine production in LPL KD microglia. Ray biotech mouse neuro array was used to determine differences in chemokine expression in media from **(A)** BV-2 202 or **(B)** BV-2 553 cells. **(C–F)** Comparative gene expression between control (BV-2 202) and LPL KD (BV-2 553) cells (*N* = 4 per group). **(G)** *Ex vivo* brain slices following incubation with conditioned media (CM) from either control (WT CM) or LPL KD (KD CM) for 24 h, characterizing Plp-eGFP MAG, neurofilament or Iba1 expression (*N* = 3 per group). ***P < 0.001 vs. CONT.

To further understand the inflammatory phenotype of BV-2 cells lacking LPL, the relative abundance of cytokines and chemokines in CM from LPL KD and control cells was determined. Monocyte chemoattractant protein (MCP-1), which is associated with microglial migration and proliferation, but not pro-inflammation (Hinojosa et al., 2011), was detected at a similar abundance in both control (Figure 3A), and LPL KD (Figure 3B) cells. Similarly, macrophage colony stimulating factor (M-CSF) was secreted at similar levels by both cell lines (Figures 3A,B). In contrast, insulin-like growth factor 1 (IGF-1), which is thought to play a role in the trophic support and development of neurons, was only detected in CM from control cells (Figures 3A,B). Since these findings were only semi-quantitative, we also used real-time PCR to quantify the level of IGF-1 gene expression in LPL KD vs. control cells. IGF-1 mRNA was significantly reduced (−12 fold) in LPL KD cells (Figure 3C). The expression of additional genes associated with a pro-inflammatory phenotype was also measured. Macrophage inflammatory protein (MIP)-1alpha (CCL3), associated with neuro-inflammation in the CNS (Zhu et al., 2016), was increased (+6.7 fold) in LPL KD cells (Figure 3D) compared to control. However, the expression of IL-12 and IL-10 were not significantly different between LPL KD and control cells (Figures 3E,F). To establish whether microglia-CM from either control cells or cells lacking LPL could elicit an inflammatory response *ex vivo*, brain slices were cultured with CM for 24 h. Histological analysis revealed that while myelin markers remained unchanged between groups, the expression of Iba1, associated with classically activated microglia, was increased in slices treated with CM from LPL KD cells (Figure 3G).

**Figure 4:**
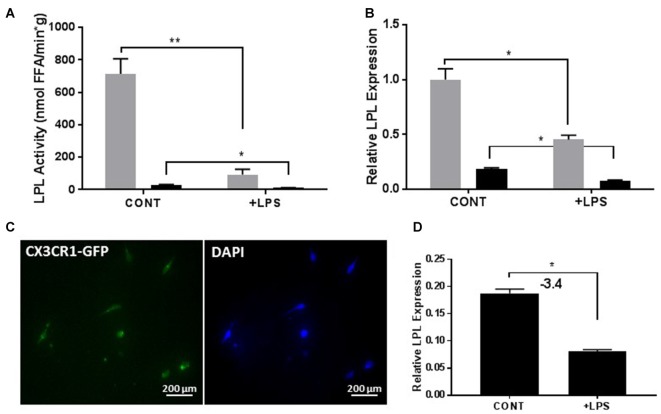
Lipopolysaccharide (LPS) activation reduces LPL expression and activity in both immortalized cells (*N* = 4 per group; **A,B**) and primary microglial cells from adult brain (**C,D**; *N* = 3 per group). *P < 0.05 vs. CONT. **P < 0.01 vs. CONT.

It is well established that LPS can stimulate an inflammatory response in BV-2 microglia (Orihuela et al., 2016; Sun et al., 2017). Since our data suggest that LPL is a feature of reparative microglia, we hypothesized that LPS-mediated microglial polarization would influence LPL dynamics. Following LPS treatment, both the enzymatic activity (Figure 4A) and mRNA expression (Figure 4B) of LPL were significantly reduced. Although the profile of the BV-2 microglia resembles that of primary microglia, it is important to assess the role of LPL in primary microglial cultures. Therefore, to test the dynamics of LPL following microglial polarization, primary microglia were isolated from adult brain (Figure 4C) and incubated with LPS for 24 h. Following LPS stimulation LPL gene expression was significantly reduced (Figure 4D).

**Figure 5:**
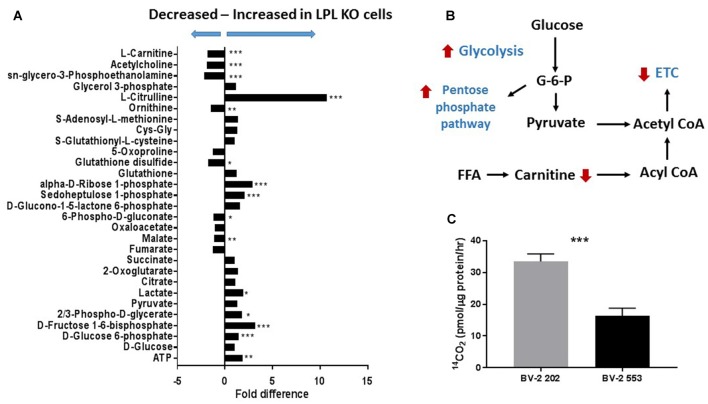
Glycolysis is increased and fatty acid oxidation (FAO) is decreased in BV-2 microglial cells lacking LPL. **(A)** Global metabolomics in control and LPL KD BV-2 microglia (*N* = 3 per group). **(B)** Schematic representation of metabolic reprogramming following depletion of LPL in microglia. **(C)** FAO in control vs. LPL KD BV-2 microglia (*N* = 6 per group). *P < 0.05 vs. CONT. **P < 0.01 vs. CONT. ***P < 0.001 vs. CONT.

Although microglial activation states are complex, it is likely that microglial polarization resembles that of M1/M2 macrophages (Orihuela et al., 2016). In brief, metabolic functions in reparative (M2-like) macrophages is characterized by FA oxidation (FAO; Odegaard and Chawla, 2011), comparatively lower glucose consumption as compared to M1 macrophages, and arginine metabolism is shifted towards ornithine and polyamines (Mills et al., 2000). By contrast, acutely activated M1-like macrophages are shifted towards increased glycolysis (Rodríguez-Prados et al., 2010), activation of the pentose phosphate pathway (PPP; Haschemi et al., 2012), and the conversion of L-arginine to L-citrulline by iNOS. To further understand the involvement of microglial LPL in the metabolic reprogramming associated with polarization we performed global metabolomics in control and LPL KD BV-2 cells (Figure 5A). We found that glucose metabolism, PPP metabolism and L-citrulline were significantly increased in LPL KD cells (Figure 5A), consistent with the metabolic changes associated with M1-like polarization (Figure 5B). We also found that FAO was increased in control cells vs. LPL KD cells, consistent with the metabolic profile of M2-like macrophages (Figure 5C).

### Lipoprotein Lipase Is Involved in Microglial Lipid Uptake

**Figure 6:**
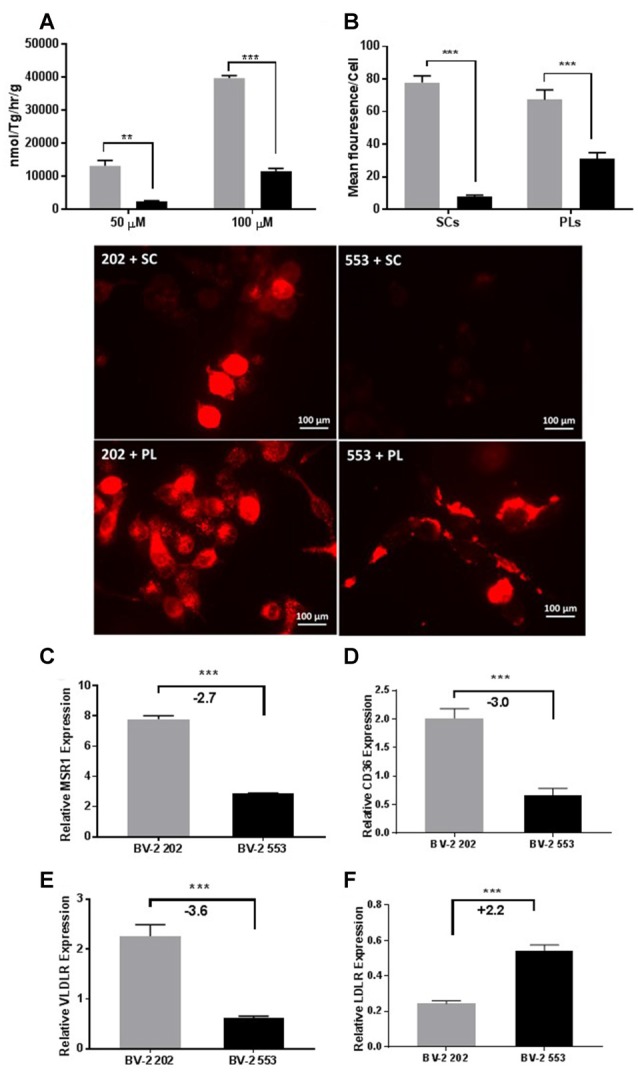
LPL is involved in lipid and lipoprotein uptake in microglia. **(A)** Intracellular uptake of radiolabeled synthetic lipid vesicles containing 50 or 100 μM Triolein Tg. **(B)** Uptake/phagocytosis of 1′-dioctadecyl-3,3,3′3′-tetramethylindocarbocyanine perchlorate (DiI) labeled synthetic chylomicrons (SC) and phospholipid liposomes (PLs; *N* = 3 per group). **(C–F)** Expression of scavenger and lipoprotein receptors in BV-2 202 vs. 553 cells (*N* = 4 per group). **P < 0.01 vs. CONT. ***P < 0.001 vs. CONT.

Several studies have implicated LPL in phagocytosis and repair. However, to date the precise mechanisms have not been determined. To test whether microglial LPL is involved in the uptake of free fatty acids (FFA), either control or LPL KD cells were incubated with synthetic TG-rich chylomicrons (SCs) containing radiolabeled FFAs. After incubation, the amount of intracellular radiolabeled FFA was quantified. FFA uptake was significantly increased in LPL expressing control microglia (Figure 6A). To address whether uptake was dependent on enzymatic hydrolysis by LPL, SCs were labeled with DiI and the internalization of intact (non-hydrolyzed) DiI labeled SC was visualized by fluorescence microscopy. SC uptake was undetectable in cells lacking LPL (Figure 6B). Since low-density lipoproteins are scarce in the brain we next asked whether LPL was involved in the uptake of myelin associated phospholipids (PLs). BV-2 cells were incubated with DiI labeled liposomes containing PC and PS. Although BV-2 cells lacking LPL internalized PLs, this was much greater in the control cells (Figure 6B). Taken together these data suggest that microglia LPL is involved in the uptake, and phagocytosis of lipids. Therefore, we measured the expression of factors associated with phagocytosis. We found that MSR1, CD36, and VLDLR expression was markedly reduced in LPL KD cells (Figures 6C–E); however, LDLR, which is the major importer of ApoE containing lipoproteins in the brain, was significantly increased (Figure 6F).

### Conditioned Media From LPL KD Cells Inhibits Remyelination and Repair

**Figure 7:**
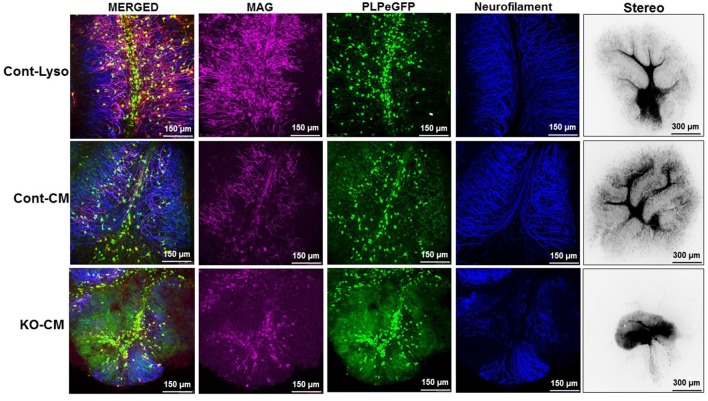
CM from LPL KD cells prevents normal remyelination processes. *Ex vivo* cerebellar slices were demyelinated by overnight treatment (17 h) with lysolecithin. The next day slices were incubated with either standard slice media, or CM and allowed to recover for 7 days.

To determine whether LPL directly plays a role in the remyelination process, *ex vivo* brain slices were demyelinated with lysolecithin and then allowed to recover in CM from LPL-secreting control cells and cells lacking LPL (Figure 7). After a 7-day recovery period, cells treated with lysolecithin, but then incubated in optimal slice media showed normal tissue morphology and expression of myelin markers. In slices treated with CM from control cells, the slice morphology was comparable to control, and there was no change in myelin marker expression. However, brain slices treated with CM from LPL KD cells showed a somewhat necrotic morphology with diffuse myelin marker staining and a significant reduction in neurofilament expression, suggesting that re-myelination was incomplete in this condition.

## Discussion

MS is a relatively common (1:1000 in susceptible populations) debilitating disease that can affect an individual for much of their life. During the RR-MS stage of the disease there is a discrete balance between de- and remyelination. In P-MS, these attempts to remyelinate fail leading to neurodegeneration and progressive disability. Understanding the dynamics of de- and remyelination may be key to identifying potential targets for MS treatments. Microglial function plays a key role in the remyelination process. The production of cytokines/chemokines from the appropriate microglial phenotype can stimulate the proliferation of OPCs to promote remyelination (Voss et al., 2012). In addition, microglial-phagocytosis of myelin debris plays in important role in the initiation of remyelination (Neumann et al., 2009). However, to date the factors involved in these processes remain elusive. Here we show for the first time that LPL, a lipid-processing enzyme with known roles in hydrolytic and non-hydrolytic lipid uptake, was a feature of an “alternatively activated” microglial phenotype associated with phagocytosis and remyelination.

In the present study we have demonstrated that LPL is increased at the initiation of remyelination in both *in vivo* and *ex vivo* model systems (Figure 1). Using the EAE model of demyelination we showed that LPL activity in the brain is significantly increased at 30 days following immunization. This time point correlates with a nadir in clinical symptoms, suggesting that LPL expression is associated with the partial remyelination observed in RR-MS. In further support of this notion, we have shown that LPL abundance and enzymatic activity is increased at the interphase of de- and remyelination in an *ex vivo* model of demyelination. These observations are consistent with findings from a cuprizone model of de- and remyelination in which LPL was identified as a key feature of a microglial phenotype that actively supports remyelination (Olah et al., 2012). Since cuprizone models of de- and remyelination have been used to identify the central role of debris clearance in these processes (Voss et al., 2012), these data suggest that LPL is a feature of a scavenging microglial phenotype that promotes regeneration.

Microglia play an important role in remyelination and regeneration process through their ability to clear debris, and their expression and secretion of cytokines and growth factors. Our data robustly show that cells lacking LPL polarize to a pro-inflammatory phenotype characterized by the expression of iNOS, inflammatory cytokines, and a metabolic shift towards glycolysis—similar to that of M1-like macrophages. In contrast, cells that express LPL maintain an alternately activated phenotype that is characterized by elevated Arg1 expression, IGF-1 production and increased FAO. The switch in expression between Arg1 and iNOS is particularly indicative of microglial polarization, since both Arg1 and iNOS use the same substrate (arginine) to either produce polyamines for wound healing and matrix deposition (Arg1; Munder, 2009), or nitric oxidase production contributing to inflammation (iNOS; Morris, 2007). Thus, Arg1 vs. iNOS represents a useful switch to an M2 vs. M1 phenotype. In the present study we show that the loss of LPL markedly increases iNOS expression while reducing Arg1 expression, suggesting a polarization towards an M1-like phenotype (Figure 2). In further support we show that L-citrulline, a product in the synthesis of NO by iNOS (Chaturvedi et al., 2007), was markedly increased in cells lacking LPL (Figure 5). Although defined phenotypic boundaries likely do not represent the full microglial spectra observed in complex disease states such as MS, the expression of these genes serve as useful categorical markers. In addition, the expression of chemokines and growth factors are also useful markers of microglial status. Therefore, it is of interest that microglial cells lacking LPL have significantly reduced IGF-1 secretion and expression (Figure 3), which has been classically associated with M2-like macrophages. Recent studies have shown that IGF-1 plays a key role in the CNS, where it exerts a trophic effect on neurogenesis and neuronal survival (Russo et al., 2005). Moreover, microglia are an important source of IGF-1, and its production is linked to microglial inflammatory status (Suh et al., 2013). Interestingly, reduced IGF-1 has been observed in AD (Alvarez et al., 2007), and in patients with MS (Shahbazi et al., 2017). Recent studies using rodent models of local lysolecithin induced demyelination have shown that intrathecal IGF-1 administration promotes remyelination (Hlavica et al., 2017). Taken together, these observations suggest that LPL expression promotes a microglial polarization towards an anti-inflammatory and reparative phenotype associated with IGF-1 production, a key factor that links microglial function to repair and remyelination in MS.

Our findings prompt the question; how does modulated LPL expression mediate microglial polarization? Since LPL depletion is associated with a shift towards glycolysis and away from FAO, it is likely that LPL loss causes a shift in lipid availability, substrate metabolism, and polarization towards an M1-like classically activated phenotype. In contrast, cells harboring endogenous LPL levels may have sufficient lipid-uptake, facilitating the shift towards FAO that is associated with M2-like alternatively activated and reparative microglia. Precisely why increased FAO is important for reparative microglial remains to be tested, but one may speculate that FAO provides the additional energy supply required for long-term reparative processes.

In support of the notion that LPL facilitates microglial lipid uptake, we have also shown that microglia lacking LPL are less able to take up FFAs from synthetic chylomicrons than cells expressing endogenous levels of LPL (Figure 6). These data are consistent with the canonical function of LPL—the hydrolysis of TG-rich lipoproteins—and suggests that microglial LPL may play a similar role in the CNS. Since TG-rich lipoproteins are scarce in the CNS, we hypothesized that LPL may facilitate the up-take of myelin-associated lipids. The phospholipids PC and PS are major components of myelin with pleotropic roles in the pathogenesis of MS (Ho et al., 2012). In the present study we show that LPL is involved in the uptake of PC and PS containing liposomes (Figure 6), highlighting the role of microglial LPL in myelin phagocytosis. It is well established that the phagocytosis and clearance of myelin debris is important for the remyelination process (Neumann et al., 2009). For example, ineffective microglial function in CXC3CR1 knockout mice has been shown to impair debris clearance and subsequent remyelination (Lampron et al., 2015). In addition, many recent reports have highlighted the potential role of microglial-LPL in phagocytosis and myelin clearance (Olah et al., 2012; Ma et al., 2013; Keren-Shaul et al., 2017). Although the precise mechanism regarding the mode of phagocytosis remains to be determined, recent data from mice deficient in Triggering receptor expressed on myeloid cells 2 (TREM 2)—a microglial immunoreceptor associated with phagocytosis and whose loss-of-function mutations in humans cause presenile dementia—have shown that LPL is markedly reduced in this model, suggesting that LPL is involved in TREM 2-mediated phagocytosis (Cantoni et al., 2015). In addition, the mechanism underlying the role of LPL in phagocytosis may be similar to its non-hydrolytic, or endocytotic bridging function. LPL can facilitate lipid-uptake through simultaneous interaction with lipoproteins, heparin sulfate proteoglycans (HSPG), or cell surface receptors such as lipoprotein receptors (Makoveichuk et al., 2004). Conversely, it is well established that lipoprotein receptors have a duel function in immune cells, and may also serve as scavenging receptors to facilitate phagocytosis. For example, the low density lipoprotein receptor-related protein-1 (LRP1) functions as a scavenger receptors for myelin debris (Fernandez-Castaneda et al., 2013). It is thus plausible to speculate that LPL may simultaneously bind to lipid-rich myelin debris and cell surface lipoprotein receptors to expedite myelin clearance and remyelination.

Collectively, our observations suggest that microglial LPL exerts simultaneous benefits for repair and remyelination. On one hand the presence of LPL is associated with a reparative microglial phenotype, the production of important neurotropic factors, and the maintenance of appropriate substrate availability for metabolic polarization towards and M2-like phenotype. In addition, microglial LPL is directly involved in the internalization of myelin associated lipids. In summary, our findings highlight the importance of LPL in demyelinating disorders and are the first to demonstrate that microglial-LPL is directly involved in reparative processes needed for remyelination. We anticipate that the role of LPL in debris clearance and uptake may be exploited to develop future therapies for demyelinating disorders such as MS, and also primary neurodegenerative disease.

## Author Contributions

KDB, WBM and RHE designed the experiments. KDB, SG, KG and AMC performed the experiments; analyzed the data. KDB, KEB, WBM and RHE interpreted data. KDB wrote the manuscript and all authors edited the manuscript.

## Conflict of Interest Statement

The authors declare that the research was conducted in the absence of any commercial or financial relationships that could be construed as a potential conflict of interest.
